# Supplementary material for: Describing the fecal metabolome in cryogenically collected samples from healthy participants
Source: Sci Rep. 2020 Jan 21;10:885. doi: 10.1038/s41598-020-57888-w (PMC6972823; doi:10.1038/s41598-020-57888-w)
Supplement: Supplementary file 1 — Supplementary Information. [file 41598_2020_57888_MOESM1_ESM.pdf]

Supporting information to:

Describing the fecal metabolome in cryogenically collected samples from healthy participants.

Authors: Kajetan Trošt[1*], Linda Ahonen[1*$], Tommi Suvitaival[1], Nina Christiansen[1], Trine Nielsen[4], Maja Thiele[2,3], Suganya Jacobsen[2,3], Aleksander Krag[2,3], Peter Rossing[1,7], Torben Hansen[4], Lars Ove Dragsted[5#], Cristina Legido-Quigley[1,6#]

[1] Steno Diabetes Center Copenhagen, Gentofte, Denmark

[2]Department of Gastroenterology and Hepatology and Odense Patient Data Exploratory Network (OPEN), Odense University Hospital, Odense, Denmark

[3]Institute for Clinical Research, University of Southern Denmark, Odense, Denmark

[4]The Novo Nordisk Foundation Center for Basic Metabolic Research, Faculty of Health and Medical Sciences, University of Copenhagen, Copenhagen, Denmark

[5]Department of Nutrition, Exercise and Sports, Faculty of Science, University of Copenhagen, Copenhagen, Denmark

[6]Institute of Pharmaceutical Science, London, King's College London, UK

[7]Faculty of Health and Medical Sciences, University of Copenhagen, Copenhagen, Denmark

[$]Current address: Biosyntia ApS, Copenhagen, Denmark

*Shared first authorship

[#]Shared last authorship

Corresponding author: Cristina Legido-Quigley; email address: cristina.legido.quigley@regionh.dk

List of supplementary figures and tables:

Figure S1: Simplified metabolomic workflow

Methods: Chemicals, sample preparation and instrumental analyses for GC-GC-MS and Lipidomics.

Figure S2: Postprocessing data workflow

Table S1. Selected set of polar molecules with relative standard deviation lower than 30% in pooled samples.

Table S2. Selected set of lipids with relative standard deviation lower than 30% in pooled samples.

Supplementary Figure S1: Simplified metabolomic workflow

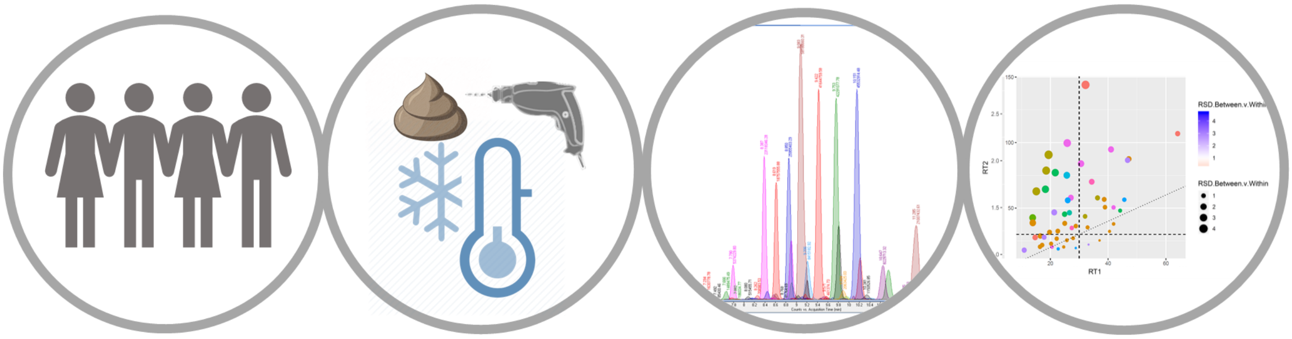

Methods: Chemicals, sample preparation and instrumental analyses for GC-GC-MS and Lipidomics

Chemicals

Water (H_2O), methanol (MeOH), acetonitrile (ACN), isopropanol (IPA) of LC-MS grade were purchased from Honeywell (Morris Plains, NJ, USA). Hexane, sodium chloride (NaCl), formic acid (HCOOH) and chloroform ($CHCl_3$) of reagent grade and ammonium acetate of LC-MS grade were purchased from Sigma-Aldrich (Steinheim, Germany). The derivatization agents methoxyamine hydrochloride (MeOX; TS-45950) and N-methyl-N-trimethylsilyltrifluoroacetamide (MSTFA) were purchased from Thermo Scientific and Sigma-Aldrich, respectively. The group-specific internal standards used for analyzing the polar metabolites were purchased from Sigma-Aldrich and they were as follows: DL-Valine-d_8, Heptadecanoic acid-d_{33}, Succinic acid-2,2,3,3-d_4, L-glutamic acid-2,3,3,4,4-d_5, trans Cinnamic acid-d_7 and Uric acid-1,3-$^{15}N_2$. Syringe standard 4,4-dibromooctafluorobiphenyl and retention index standards: Undecane (C11), Pentadecane (C15), Heptadecane (C17), Heneicosane (C21) and Pentacosane (C25) were also purchased from Sigma-Aldrich. Quality control of the lipidomics analyses was performed by adding a standard solution containing nine different lipid standards representing the different lipid classes to each sample. The following of these compounds were purchased from Avanti Polar Lipids, Inc. (Alabaster, AL, USA): 1,2-diheptadecanoyl-*sn*-glycero-3-phosphoethanolamine (PE(17:0/17:0)), N-heptadecanoyl-D-*erythro*-sphingosylphosphorylcholine (SM(d18:1/17:0)),N-heptadecanoyl-D-*erythro*-sphingosine (Cer(d18:1/17:0)), 1,2-diheptadecanoyl-*sn*-glycero-3-phosphocholine (PC(17:0/17:0)), 1-heptadecanoyl-2-hydroxy-*sn*-glycero-3-phosphocholine (LPC(17:0)), 1-palmitoyl-d31-2-oleoyl-*sn*-glycero-3-phosphocholine (PC(16:0/d31/18:1)), 1-hexadecyl-2-(9Z-octadecenoyl)-sn-glycero-3-phosphocholine (PC(16:0e/18:1(9Z))), 1-(1Z-octadecenyl)-2-(9Z-octadecenoyl)-sn-glycero-3-phosphocholine (PC(18:0p/18:1(9Z))), 1-octadecanoyl-sn-glycero-3-phosphocholine (LPC(18:0)), 1-(1Z-octadecenyl)-2-docosahexaenoyl-*sn*-glycero-3-phosphocholine (PC(18:0p/22:6)) and 1-stearoyl-2-linoleoyl-*sn*-glycerol (DG(18:0/20:4)). 1,2-dimyristoyl-*sn*-glycero-3-phospho(choline-d13) (PC(14:0/d13)), 1,2,3-triheptadecanoylglycerol (TG(17:0/17:0/17:0)) and 3β-hydroxy-5-cholestene 3-linoleate (ChoE(18:2)) was purchased from Sigma-Aldrich and tripalmitin-1,1,1-13C3 (TG(16:0/16:0/16:0)-13C3), trioctanoin-1,1,1-13C3 (TG(8:0/8:0/8:0)-13C3) and 1-palmitoyl-2-hydroxy-sn-Glycero-3pPhosphatidylcholine (LPC(16:0)) from Larodan AB (Solna, Sweden). Additionally, calibration curves (at concentration levels of 100, 500, 1000, 1500, 2000 and 2500 ng mL^{-1} for the quantification of lipids were prepared using 1-hexadecyl-2-(9Z-octadecenoyl)-sn-glycero-3-phosphocholine (PC(16:0e/18:1(9Z))), 1-(1Z-octadecenyl)-2-(9Z-octadecenoyl)-sn-glycero-3-phosphocholine (PC(18:0p/18:1(9Z))), 1-octadecanoyl-sn-

glycero-3-phosphocholine (LPC(18:0)), 1-(1Z-octadecenyl)-2-docosahexaenoyl-*sn*-glycero-3-phosphocholine (PC(18:0p/22:6)), 1-stearoyl-2-arachidonoyl-*sn*-glycero-3-phosphoinositol (PI(18:0/20:4)) and 1-stearoyl-2-linoleoyl-*sn*-glycerol (DG(18:0/18:2)) from Avanti Polar Lipids, Inc., 1-Palmitoyl-2-Hydroxy-sn-Glycero-3-Phosphatidylcholine (LPC(16:0)) from Larodan, and 1,2,3-Triheptadecanoylglycerol (TG(17:0/17:0/17:0)) and 3β-Hydroxy-5-cholestene 3-linoleate (ChoE(18:2)), 3β-Hydroxy-5-cholestene 3-oleate (ChoE(18:1(9Z))), 5-Cholesten-3β-yl octadecenoate (ChoE(18:0)) and 5-Cholestene 3-palmitate (ChoE(16:0)) from Sigma-Aldrich.

Sample preparation

The method used for the analysis of polar metabolites was originally developed for the analysis of plasma and cerebrospinal fluid[S1] and modified to fit the specific matrix (i.e. fecal samples) and the target analytes. An internal standard mixture consisting of 25 mg L^{-1} DL-Valine-d_8, 160 mg L^{-1} Heptadecanoic acid-d_{33}, 25 mg L^{-1} Succinic acid-d_4, 100 mg L_{-1} L-glutamic acid-d_5, 100 mg L^{-1} trans-Cinnamic acid-d_7 and 100 mg L^{-1} Uric acid-$^{15}N_2$ was created by dissolving the analytes in methanol. After this, 20mg of each homogenized sample was mixed with 600 µL of H_2O and 60 µL of the internal standard mixture. Samples were vortex-mixed and sonicated for 5 minutes after which they were incubated on ice for 30 min and centrifuged (9400 × g, 5 min, 4 °C). The supernatants were filtered through Millipore Millex PVDF syringe filters with diameter of 4 mm and pore size of 0.45 µm. Finally, 150 µL of the filtered extracts were transferred to glass vials and evaporated to dryness before further analysis. The samples were derivatized using a previously described MeOX:MSTFA procedure.[31] Here, the derivatization converts the reactive biological groups into trimethylsilyl derivates, which increase the volatility of the biomolecules. The derivatization was performed automatically using a MultiPurpose Sampler 2 (MPS2, Gerstel; Mülheim an der Ruhr, Germany) with two robotic hands. First, 25 µL of MeOX was added to each sample after which they were incubated and shaked for one hour at 45°C. Then, 25 µL of MSTFA (including a set of n-alkanes as retention index standards at 8 mg L^{-1}) was added to each sample and the samples were again incubated and shaked at 45 °C for one hour. Finally, before injecting the sample for analysis, 50 µL of the injection standard 4,4'-dibromooctafluorobiphenyl (9.8 mg L^{-1} in hexane) was added to each sample.

Analysis of metabolites by GC×GC-MS

Sample analysis

The polar metabolites were analyzed using a Pegasus 4D (LECO; Saint Joseph; USA) system, which combines two-dimensional chromatographic separation with time-of-flight (TOF) mass spectrometric detection. A volume of 1 µL of the derivatized sample extract was injected into the chromatographic system Chromatographic separation was then achieved using a system of two columns guarded by a retention gap column (1.7m, 0.53 mm ID, FS deactivated) from Agilent Technologies (Santa Clara, CA, USA). The primary separation was achieved using a 10 m × 0.18 mm I.D. Rxi-5 ms (Restek Corp., Bellefonte, PA, USA) column and the secondary column was a 1.5 m × 0.1 mm I.D. BPX-50 (SGE Analytical Science, Austin, TX, USA). The temperature gradient used for separating the analytes was as follows: 50 °C (2 min), 7°C/min to 240°C, 25°/min to 300 °C (3 min). The secondary oven temperature was set to be 20 °C higher than primary oven temperature. The modulator cycle was set to 4s, producing narrow chromatographic peaks of approximately 0.2s. Hence, the speed of the detector was set to 100 Hz. The ChromaTOF software (version 4.32; LECO Corporation, St. Joseph, USA) was used for all data acquisition as well as for processing of the raw data. The default peak picking criteria for the raw data processing was set to 15s in the first dimension and 0.2 in second

dimension and the signal to noise ratio was set to 100. The NIST14 spectral library was used for potential identification of metabolites and the created text files were exported for the further data processing.

Lipidomics analyses

Sample preparation

Previously published lipidomics procedures,[S2,S3] which have been validated for plasma samples, were used for the fecal lipidomics with some modifications. The 40 drilled fecal samples (50 mg of fecal slurry) were prepared using a modified Folch-extraction procedure.[S4] To ensure high quality of data, blank samples and a pool of all fecal samples were prepared using the same protocol and they were analyzed along with the actual samples. Briefly, 10 µL of 0.9% NaCl, 92 µL of CHCl3:MeOH (2:1, v/v) and 28 µL of a 10 µg/mL internal standard solution (containing PE(17:0/17:0), SM(d18:1/17:0), Cer(d18:1/17:0), PC(17:0/17:0), LPC(17:0), PC(16:0/d31/18:1), PC(14:0/d13), TG(16:0/16:0/16:0)-13C3 and TG(8:0/8:0/8:0)-13C3) were added to each fecal sample. The samples were vortex mixed and incubated on ice for 30 min after which they were centrifuged (9400 × *g*, 3 min, 4 °C). Finally, 60 µL from the lower layer of each sample was then transferred to a glass vial with an insert and 60 µL of $CHCl_3$:MeOH (2:1, v/v) was added to each sample. All samples were stored in -80 °C until analysis.

Sample analysis

The samples were analyzed using an UHPLC-Q-TOF-MS, which has been presented in detail previously.[36] Briefly, the UHPLC system was a 1290 Infinity system from Agilent Technologies. The system was equipped with a multisampler (maintained at 10 °C), a quaternary solvent manager and a column thermostat (maintained at 50 °C). Separations were performed on an ACQUITY UPLC® BEH C18 column (2.1 mm × 100 mm, particle size 1.7 µm) by Waters (Milford, USA). The flow rate was 0.4 mL min^{-1} and the injection volume was 1 µL. H_2O + 1% NH_4Ac (1M) + 0.1% HCOOH (A) and ACN:IPA (1:1, v/v) + 1% NH_4Ac + 0.1% HCOOH (B) were used as the mobile phases for gradient elution of the analytes and the gradient was as follows: from 0 to 2 min 35-80% B, from 2 to 7 min 80-100% B and from 7 to 14 min 100% B.

The mass spectrometer coupled to the UHPLC system was a 6550 iFunnel quadrupole time of flight (Q-TOF) from Agilent Technologies. The Q-TOF was interfaced with a dual jet stream electrospray (dual ESI) ion source. Nitrogen generated by a nitrogen generator (PEAK Scientific, Scotland, UK) was used as the nebulizing gas at a pressure of 21 psi, as the drying gas at a flow rate of 14 L min^{-1} (at 193 °C) and as the sheath gas at a flow rate of 11 L min^{-1} (at 379 °C). Pure nitrogen (6.0) from Strandmøllen A/S (Klampenborg, Denmark) was used as the collision gas. The capillary voltage and the nozzle voltage were kept at 3643 and 1500 V, respectively. The reference mass solution including ions at m/z 121.0509 and 922.0098 was prepared according to instructions by Agilent and it was introduced to the mass spectrometer through the other nebulizer in the dual ESI ion source using a separate Agilent series 1290 isocratic pump at a constant flow rate of 4 mL/min (split to 1:100). The acquisition mass range was m/z 100–1700 and the instrument was run using the extended dynamic range with an approximate resolution of 30 000 FWHM measured at m/z 1521.9715. MassHunters B.06.01 (Agilent Technologies) software was used for all data acquisition and quality check. Example chromatogram is presented in Suplementary Figure S1.

Supplementary references

S1. Hartonen, M., Mattila, I., Ruskeepää, A. L., Orešič, M. & Hyötyläinen, T. Characterization of cerebrospinal fluid by comprehensive two-dimensional gas chromatography coupled to time-of-flight mass spectrometry. *J. Chromatogr. A* **1293**, 142-149 (2013).

S2. Hyötyläinen, T., Ahonen, L., Pöhö, P. & Orešič, M. Lipidomics in biomedical research-practical considerations. *Biochim. Biophys. Acta* **1862**, 800-803 (2017).

S3. O'Gorman, A. *et al.* Identification of a plasma signature of psychotic disorder in children and adolescents from the Avon Longitudinal Study of Parents and Children (ALSPAC) cohort. *Transl. Psychiatry* **7**, e1240 (2017).

S4. Folch, J., Lees, M. & Sloane Stanley, G. H. A simple method for the isolation and purification of total lipides from animal tissues. *J. biol. Chem.* **226**, 497-509 (1957).

Supplementary Figure S2: Postprocessing data workflow

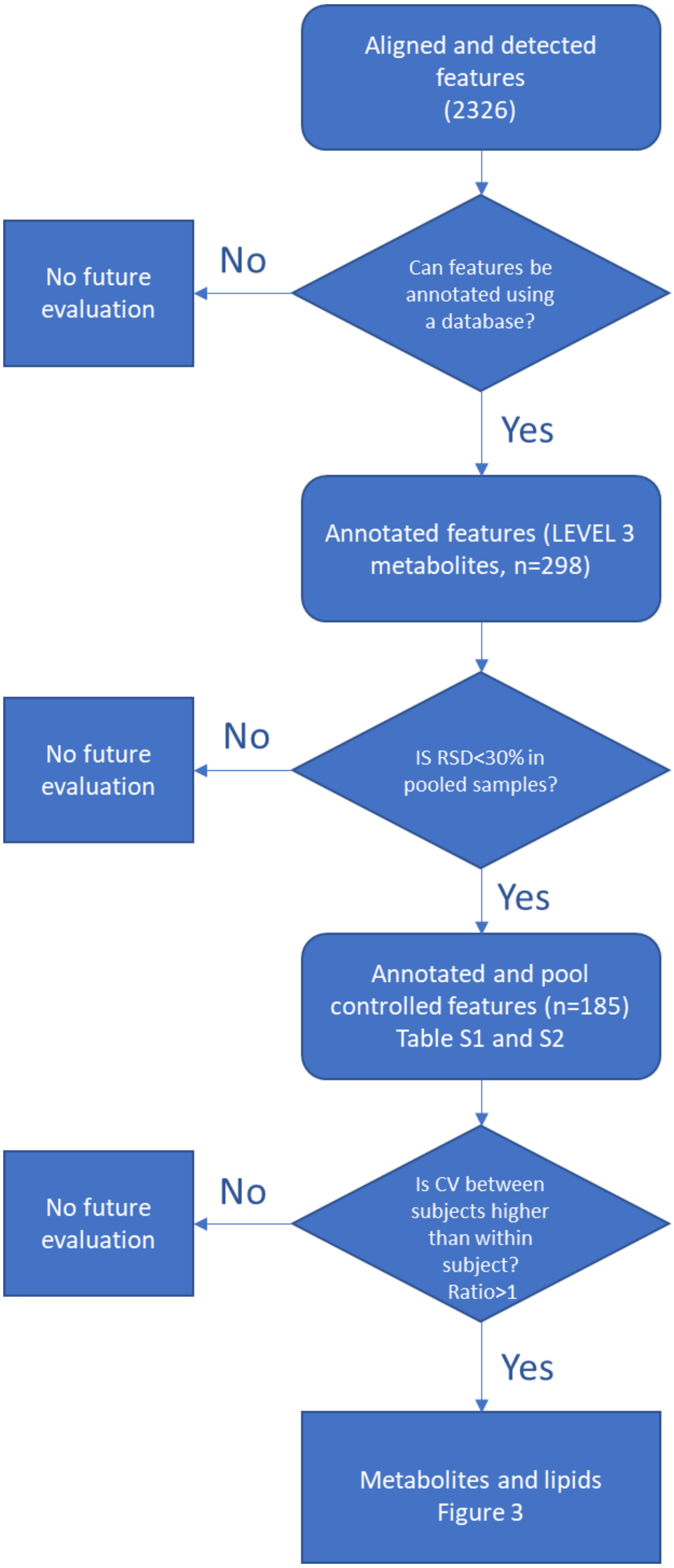

Supplementary Table S1. Selected set of polar molecules with relative standard deviation lower than 30% in pooled samples. *possible contaminant

	Name	CV - pooled sample (%)	CV Between Subjects (%)	CV Within Drills (%)	Ratio (CV between /CV within)	feature-wise F-test	adj.P.Val
**BENZENE DERIVATES**							
	3-Hydroxyphenylacetic acid	4	107	68	1.6	6	8.26E-05
	Tyramine	8	98	44	2.2	18	7.12E-10
	3-Phenyllactic acid	9	79	19	4.2	112	3.47E-20
	3-Phenylpropionic acid	10	63	15	4.2	215	1.19E-23
	4-Hydroxyphenyllactic acid	12	91	19	4.7	142	1.53E-21
	3-Hydroxyphenylpropionic acid	12	58	36	1.6	32	7.31E-13
	4-Hydroxybenzeneacetic acid	13	45	64	0.7	7	9.19E-06
	Phtalic acid*	20	92	38	2.4	16	2.51E-09
	Benzeneacetic acid	24	44	32	1.4	24	2.34E-11
	3,4-Dihydroxyhydrocinnamic acid	24	95	52	1.9	7	1.36E-05
**INDOLS**							
	3-Indoleacetic acid	20	75	26	2.9	51	1.22E-15
	3-Indolepropionic acid	23	74	45	1.6	9	1.59E-06
**CARBOXYLIC ACIDS**							
	Fumaric acid	8	77	67	1.2	8	3.73E-06
	Maleic acid	10	20	21	1.0	67	3.73E-17
	Lactic Acid	11	66	71	0.9	8	3.66E-06
	2-Hydroxybutyric acid	14	84	31	2.7	92	4.54E-19
	Citric acid	15	51	37	1.4	13	2.79E-08
	Citramalic acid	22	46	30	1.6	12	7.95E-08
	3-Hydroxybutyric acid	25	58	27	2.1	83	2.10E-18
	Tricarballylic acid	28	100	26	3.9	25	1.37E-11
	Succinic acid	30	40	49	0.8	15	7.95E-09
	2,4-Dihydroxybutyric acid	30	58	26	2.2	11	1.35E-07
**FATTY ACID DERIVATES**							
	Pentadecanoic acid	3	48	44	1.1	21	1.14E-10
	Palmitic Acid	3	33	49	0.7	12	1.20E-07
	Azelaic acid	4	39	66	0.6	10	3.53E-07
	3-Hydroxyisovaleric acid	6	54	33	1.6	6	6.42E-05
	5-Hydroxyhexanoic acid	7	45	25	1.8	51	1.22E-15
	Stearic acid	9	53	44	1.2	18	5.93E-10
	Suberic acid	9	51	67	0.8	9	1.66E-06
	Arachidic acid	12	34	54	0.6	9	1.69E-06
	Methylsuccinic acid	14	64	18	3.5	201	2.11E-23
	2-Hydroxyisocaproic acid	15	77	22	3.6	65	4.63E-17
	Linolenic acid	15	144	83	1.7	9	2.33E-06
	Hexanoic acid	17	89	68	1.3	7	1.22E-05
	Sebacic acid	18	52	50	1.1	13	3.29E-08
	Nonanoic acid	19	69	62	1.1	8	6.00E-06
	Dodecanoic acid	28	182	80	2.3	4	0.00279
	Decanoic acid	30	86	63	1.4	5	0.000263
	Myristic acid	30	87	70	1.2	6	9.34E-05

**AMINOACIDS**							
	Isoleucine	6	27	22	1.2	79	3.53E-18
	5-Oxoproline	7	29	17	1.7	150	1.02E-21
	Leucine	7	20	25	0.8	64	5.21E-17
	Glycine_iso1	8	21	16	1.3	121	1.21E-20
	Alanine	9	39	14	2.8	148	1.07E-21
	Tyrosine_iso1	10	22	17	1.3	144	1.42E-21
	Tyrosine_iso2	12	36	29	1.3	41	2.55E-14
	Acetyl-Lysine	13	26	26	1.0	45	6.19E-15
	Ornithine	13	32	20	1.6	104	8.64E-20
	Valine	13	23	20	1.1	102	1.21E-19
	Aspartic acid_iso1	16	33	23	1.4	80	3.00E-18
	Threonine_iso1	16	27	28	1.0	47	3.76E-15
	Serine_iso1	18	66	65	1.0	9	2.28E-06
	Threonine_iso2	19	60	68	0.9	8	4.53E-06
	Tryptophan_iso1	20	35	33	1.1	32	6.46E-13
	Methionine	20	65	64	1.0	9	1.83E-06
	Phenylalanine_iso1	20	67	63	1.1	9	2.55E-06
	Aspartic acid_iso2	22	41	43	0.9	19	3.12E-10
	Tryptophan_iso2	22	51	39	1.3	21	1.42E-10
	Serine_iso2	22	25	37	0.7	28	3.10E-12
	Proline	22	38	42	0.9	26	1.10E-11
	Asparagine	23	122	56	2.2	8	2.85E-06
	Glycine_iso2	23	38	25	1.5	69	2.66E-17
	Phenylalanine_iso2	24	25	44	0.6	19	3.30E-10
	4-Aminobutanoic acid	27	65	68	1.0	9	1.59E-06
**AMINES**							
	Ethanolamine_iso_1	7	28	15	1.9	129	4.80E-21
	Putrescine	22	144	32	4.5	65	4.96E-17
	Ethanolamine_iso_2	26	31	31	1.0	20	2.73E-10
	Cadaverine	30	107	64	1.7	27	6.09E-12
**PURINE DERIVATES**							
	Inosine	15	56	26	2.2	50	1.52E-15
	Hypoxanthine	20	19	23	0.8	65	4.63E-17
	2-Deoxyinosine	28	56	46	1.2	20	2.05E-10
**PYRIMIDINE DERIVATES**							
	Uracil	7	18	11	1.6	290	2.65E-25
	Thymine	7	28	18	1.6	132	3.95E-21
	Orotic Acid	8	87	47	1.9	50	1.61E-15
	Uridine_iso1	25	47	21	2.2	95	3.24E-19
	Uridine_iso2	29	41	39	1.1	20	2.70E-10
**DIOLS**							
	1,3-Propanediol	11	43	14	3.1	164	3.52E-22
**GLYCEROL DERIVATES**							
	Glycerol-3-phosphate	30	47	27	1.8	56	4.08E-16
**STEROIDS**							
	Cholesterol	4	142	54	2.7	19	3.34E-10
**SUGARS**							
	Myo-Inositol	8	35	27	1.3	47	3.76E-15
	Cellobiose	22	70	34	2.0	37	9.52E-14

Supplementary Table S2. Selected set of lipids with relative standard deviation lower than 30% in pooled samples.

ID	Name	CV - pooled sample (%)	CV Between Subjects (%)	CV Within Drills (%)	Ratio (CV between /CV within)	feature-wise F-test	adj.P.Val
**DIACYLGLYCEROL LIPIDS**							
	DG(23:0)	8	87	45	1.9	34	1.74E-13
	DG(32:1)	3	64	26	2.5	87	5.08E-19
	DG(33:09)	15	85	31	2.8	44	5.70E-15
	DG(36:3)	12	68	64	1.1	6	9.12E-05
	DG(36:4)	8	104	55	1.9	8	4.34E-06
	DG(37:1)	10	51	35	1.5	7	2.02E-05
	DG(37:4)	3	79	24	3.4	41	1.60E-14
	DG(39:5)	12	60	19	3.2	39	2.42E-14
	DG(39:5)	6	54	18	3.0	44	6.43E-15
	DG(39:5)	2	58	23	2.6	76	3.21E-18
	DG(39:6)	13	64	15	4.2	102	6.28E-20
	DG(39:6)	3	39	15	2.7	56	2.04E-16
	DG(39:6)	12	66	24	2.7	102	6.37E-20
	DG(39:6)	7	54	33	1.6	41	1.39E-14
	DG(39:7)	4	110	22	4.9	133	2.33E-21
	DG(39:7)	4	65	23	2.8	81	1.23E-18
	DG(39:7)	5	99	30	3.3	94	1.89E-19
	DG(ID_109)	4	30	23	1.3	60	9.46E-17
	DG(ID_155)	12	117	59	2.0	8	3.49E-06
	DG(ID_206)	3	33	41	0.8	22	4.71E-11
	DG(ID_90)	4	30	24	1.3	43	9.02E-15
	DG(ID_98)	15	94	24	4.0	42	1.16E-14
**TRIACYLGLYCEROL LIPIDS**							
	TG(45:0)	23	38	67	0.6	7	8.82E-06
	TG(46:0)	15	80	39	2.1	28	2.10E-12
	TG(48:1)	10	93	34	2.8	6	8.44E-05
	TG(49:3)	1	16	10	1.7	70	9.49E-18
	TG(50:0)	19	44	32	1.4	29	1.32E-12
	TG(50:0)	16	91	29	3.1	149	6.01E-22
	TG(50:1)	16	49	32	1.5	26	4.79E-12
	TG(50:1)	13	58	40	1.5	13	1.79E-08
	TG(50:2)	10	53	36	1.5	18	4.82E-10
	TG(50:3)	12	34	41	0.8	28	2.10E-12
	TG(50:4)	18	59	50	1.2	10	4.39E-07
	TG(51:1)	14	145	33	4.4	25	7.02E-12
	TG(51:1)	13	152	45	3.4	31	5.09E-13
	TG(51:2)	16	68	69	1.0	7	2.86E-05
	TG(51:3)	24	33	55	0.6	12	7.00E-08
	TG(51:4)	20	73	57	1.3	5	0.000245
	TG(52:1)	8	58	43	1.3	18	4.73E-10
	TG(52:1)	18	45	48	0.9	19	1.93E-10
	TG(52:2)	14	42	39	1.1	13	1.88E-08

	TG(52:2)	16	39	40	1.0	21	5.44E-11
	TG(52:3)	5	49	36	1.4	37	5.86E-14
	TG(52:3)	13	41	39	1.0	7	8.49E-06
	TG(52:4)	4	46	35	1.3	30	8.45E-13
	TG(52:4)	5	27	34	0.8	37	5.95E-14
	TG(52:5)	5	23	38	0.6	26	4.15E-12
	TG(52:6)	2	82	71	1.2	10	5.06E-07
	TG(54:3)	16	31	40	0.8	25	7.11E-12
	TG(54:3)	6	37	36	1.0	16	2.07E-09
	TG(54:4)	8	52	54	1.0	4	0.00182
	TG(54:4)	8	31	44	0.7	19	1.94E-10
	TG(54:5)	5	34	34	1.0	30	7.72E-13
	TG(54:6)	1	37	36	1.0	42	1.04E-14
	TG(54:6)	0	41	44	0.9	19	2.41E-10
	TG(56:4)	3	69	45	1.5	23	2.04E-11
	TG(56:5)	6	42	35	1.2	29	1.44E-12
	TG(58:4)	3	90	75	1.2	5	0.000211
	TG(ID_105)	13	53	51	1.1	23	2.13E-11
	TG(ID_144)	14	36	45	0.8	25	7.11E-12
	TG(ID_146)	23	77	35	2.2	30	7.31E-13
	TG(ID_159)	21	46	79	0.6	9	1.38E-06
	TG(ID_166)	20	51	73	0.7	3	0.00482
	TG(ID_192)	13	55	37	1.5	33	3.02E-13
	TG(ID_198)	12	58	38	1.6	8	4.50E-06
	TG(ID_220)	12	104	53	2.0	8	6.30E-06
	TG(ID_246)	20	36	44	0.8	25	7.42E-12
	TG(ID_308)	7	38	39	1.0	9	1.11E-06
	TG(ID_315)	8	27	29	0.9	41	1.39E-14
	TG(ID_351)	18	74	29	2.5	52	7.37E-16
	TG(ID_57)	3	52	37	1.4	4	0.000791
	TG(ID_94)	14	64	44	1.5	4	0.000877
	TG(ID_99)	11	83	34	2.4	2	0.0271
**CERAMIDES**							
	Cer(d27:1)	8	128	36	3.5	2	0.142
	Cer(d29:1)	8	82	43	1.9	2	0.0392
	Cer(d32:0)	14	96	16	6.1	110	2.49E-20
	Cer(d33:1)	23	62	24	2.6	35	1.04E-13
	Cer(d34:0)	15	66	25	2.7	30	7.76E-13
	Cer(d34:1)	25	61	25	2.4	105	4.45E-20
	Cer(d34:1)	5	88	26	3.3	134	2.32E-21
	Cer(d35:0)	14	127	29	4.4	124	4.80E-21
	Cer(d35:1)	15	29	29	1.0	12	6.94E-08
	Cer(d35:1)	17	44	32	1.4	31	4.94E-13
	Cer(d35:2)	22	91	16	5.6	241	1.98E-24
	Cer(d36:0)	11	92	20	4.6	149	6.01E-22
	Cer(d40:2)	6	109	41	2.7	87	5.08E-19
	Cer(d42:2)	5	91	31	3.0	127	3.79E-21
	Cer(d42:2)	5	80	30	2.7	96	1.47E-19
	Cer(ID_347)	8	56	46	1.2	8	4.97E-06
	Cer(ID_368)	8	44	27	1.6	30	9.34E-13
**LYSOPHOSPHATIDYLCHOLINES**							
	LPC(16:0)	18	64	47	1.4	11	1.68E-07

	LPC(18:0)	7	61	48	1.3	22	4.71E-11
	LPC(18:1)	19	92	51	1.8	5	0.00042
**GLYCEROPHOSPHOCHOLINES**							
	PC(34:1)	8	61	44	1.4	13	2.31E-08
	PC(34:2)	11	51	54	0.9	5	0.000365
	PC(36:3)	9	107	67	1.6	4	0.00125
	PC(36:4)	13	89	85	1.1	3	0.00477
**GLYCEROPHOSPHOGLYCEROLS**							
	PG(39:4)	13	113	22	5.2	198	1.90E-23
	PG(ID_113)	15	95	14	6.9	159	3.58E-22
	PG(ID_349)	7	30	27	1.1	39	3.14E-14
**GLYCEROPHOSPHOINOSITOLS**							
	PI(ID_237)	10	108	44	2.5	75	3.86E-18
	PI(ID_62)	13	121	23	5.2	87	5.08E-19
**SPHINGOMYELINS**							
	SM(d34:1)	8	156	23	6.7	124	4.80E-21
	SM(d36:1)	4	53	23	2.4	76	3.17E-18
